# Supplementary material for: Targeting miR‐223 in neutrophils enhances the clearance of Staphylococcus aureus in infected wounds
Source: EMBO Mol Med. 2018 Aug 31;10(10):e9024. doi: 10.15252/emmm.201809024 (PMC6180296; doi:10.15252/emmm.201809024)
Supplement: Supplementary file 1 — Appendix [file EMMM-10-e9024-s001.pdf]

Appendix

Targeting *miR-223* in neutrophils enhances the clearance of *Staphylococcus aureus* in infected wounds

Maiko de Kerckhove, Katsuya Tanaka, Takahiro Umehara, Momoko Okamoto, Sotaro Kanematsu, Hiroko Hayashi, Hiroki Yano, Soushi Nishiura, Shiho Tooyama, Yutaka Matsubayashi, Toshimitsu Komatsu, Seongjoon Park, Yuka Okada, Rina Takahashi, Yayoi Kawano, Takehisa Hanawa, Keisuke Iwasaki, Tadashige Nozaki, Hidetaka Torigoe, Kazuya Ikematsu, Yutaka Suzuki, Katsumi Tanaka, Paul Martin, Isao Shimokawa, Ryoichi Mori

Table of contents

Appendix Figure S1. Flowchart for the comprehensive identification of wound-induced Ago2 bound-miRNA complexes using NGS.

Appendix Figure S2. Expression of *miR-223* in skin wound healing.

Appendix Figure S3. Flowchart for the comprehensive identification of *miR-223* target mRNAs using Strand NGS and NGS data.

Appendix Figure S4. *miR-223*$^{Y/-}$ neutrophils improve *S. aureus*-infected skin wound healing.

Appendix Table S1. Number of annotated reads at various time points in skin wound sites.

Appendix Table S2. Top 9 candidate inflammation-related miRNA reads.

Appendix Table S3. High expression of miRNA reads at various time points in skin wound sites.

Appendix Table S4. The profile of each human sample.

Appendix Table S5. List of antibodies.

Appendix Table S6. List of primers.

Appendix Table S7. Statistical analysis.

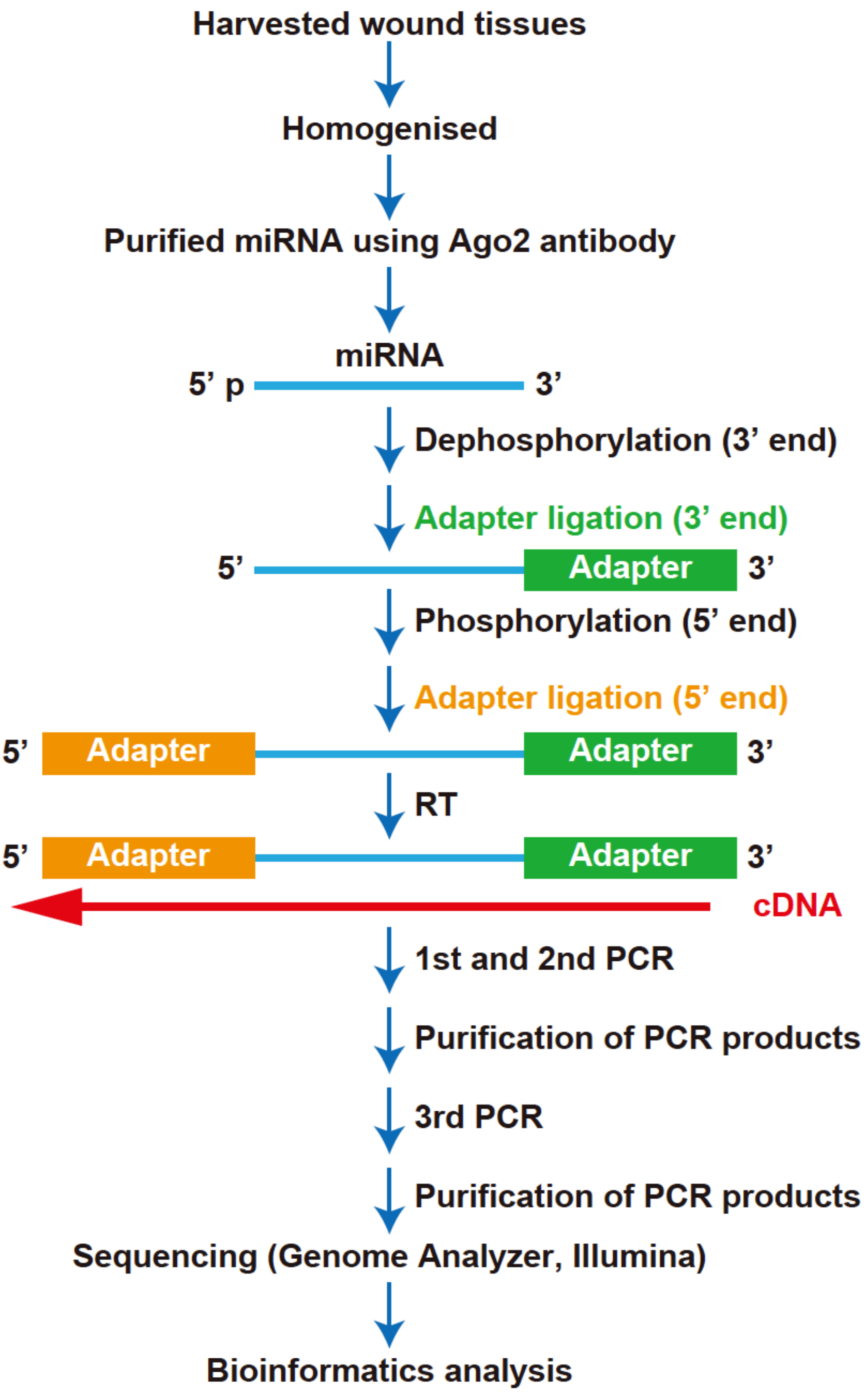

Appendix Figure S1. Flowchart for the comprehensive identification of wound-induced Ago2 bound-miRNA complexes using NGS.

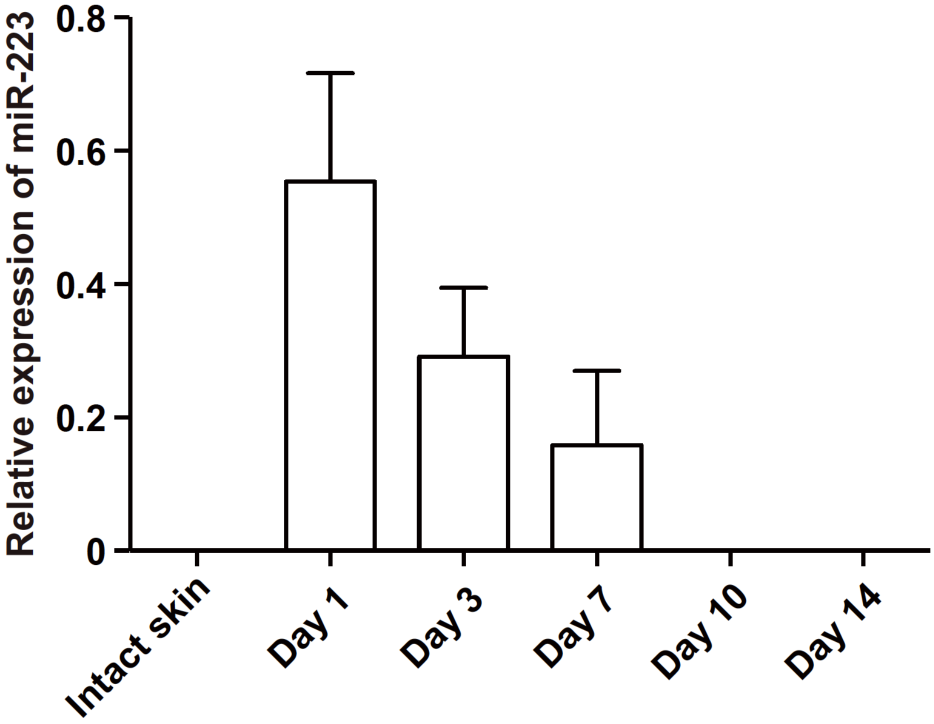

Appendix Figure S2. Expression of *miR-223* in skin wound healing.

Expression of *miR-223* in murine skin wound healing measured by qPCR relative to snoRNA202 (n = 4 - 6). Part of this results (intact, d 1, d 3, d7) are related to expression of *miR-223* corresponding to each time point in Figure 1. Data information: All values represent the mean ± SD.

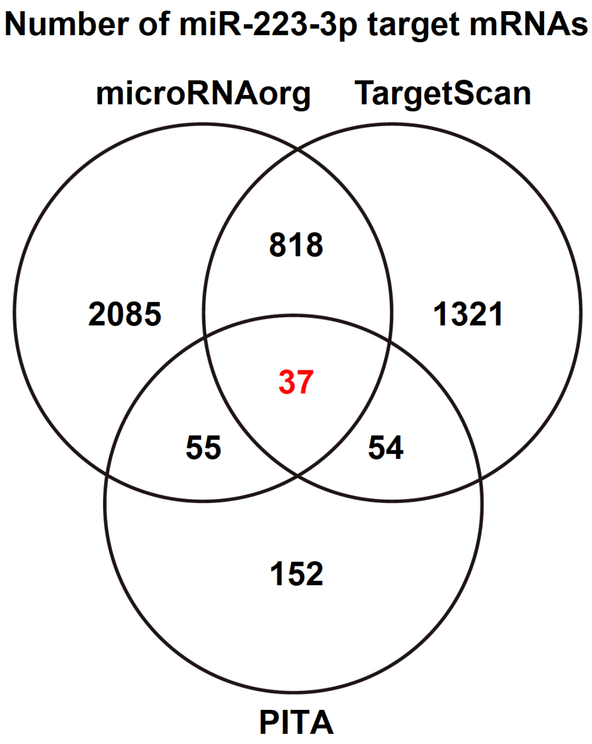

37 miR-223-3p target mRNAs

Acvr2a	*Il6*	*Slc39a1*	*Hapln2*	*Aldh1l2*
Alcam	*Nfia*	*Actr1a*	*Myh10*	*Hlf*
Axin2	*Ppp1r14b*	*Agtpbp1*	*Klhl1*	*Pkp4*
Cd247	*Rbbp6*	*Atp2b1*	*Slc16a6*	*Lrrc8d*
Ebf3	*Tgfbr3*	*Tmem178*	*Galnt7*	*Med19*
F3	*Psma6*	*Ndnf*	*Ube2q2*	
Gpam	*Pclo*	*Ttc4*	*Csrnp2*	
Has3	*Tnfrsf19*	*Tmed5*	*Frmd4a*	

Screening of miR-223-3p target genes comparison 37 miRs with NGS data

Identification of miR-223-3p target mRNA 1 d wound sites

Il6, Pclo

Appendix Figure S3. Flowchart for the comprehensive identification of *miR-223* target mRNAs using Strand NGS and NGS data.

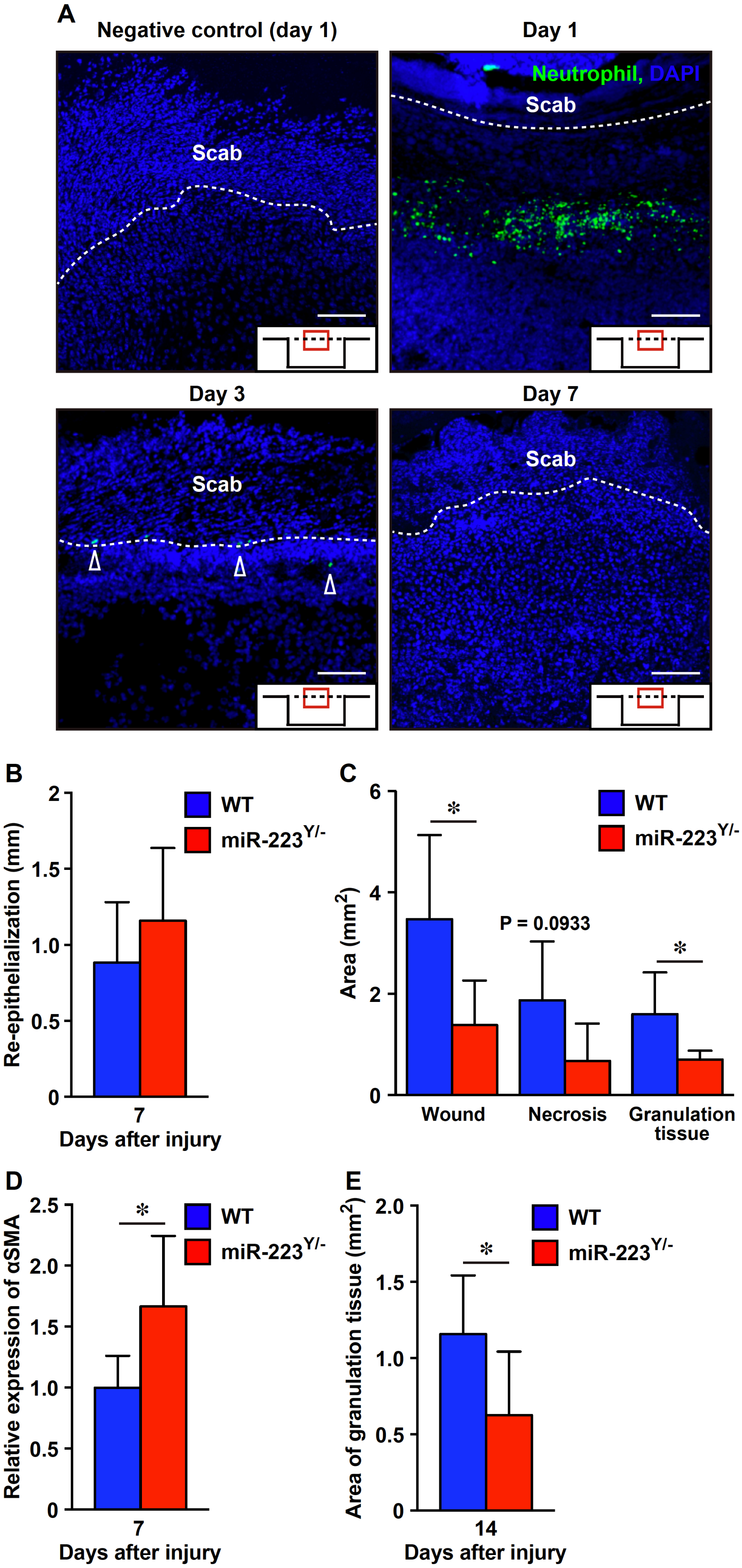
A
Negative control (day 1)
Day 1
Neutrophil, DAPI
Scab
Day 3
Day 7
B
Re-epithelialization (mm)
2
1.5
1.0
0.5
0
WT
miR-223Y/-
7
Days after injury
C
Area (mm2)
6
4
2
0
*
P = 0.0933
Wound
Necrosis
Granulation tissue
D
Relative expression of αSMA
2.5
2.0
1.5
1.0
0.5
0
E
Area of granulation tissue (mm2)
14
Days after injury

Appendix Figure S4. ***miR-223*$^{Y/-}$ neutrophils improve *S. aureus*-infected skin wound healing.**

A. Transplanted EGFP-expressing WT neutrophils in *S. aureus* skin wound sites. Representative images of EGFP-expressing WT neutrophils in *S. aureus* skin wound sites at various timepoints after EGFP-expressing neutrophil transplantation (n = 4). Scale bar, 50 µm.

B. Measurement of the epithelial tongue at d 7 after injury in WT neutrophil-transplanted (WT) and *miR-223*$^{Y/-}$ neutrophil-transplanted (*miR-223*$^{Y/-}$) wounds (n = 5).

C. Measurement of the total wound area, necrotic lesion area, and granulation tissue area at d 7 after injury in WT neutrophil-transplanted and *miR-223*$^{Y/-}$ neutrophil-transplanted wounds (n = 5).

D. Quantification of the expression of αSMA at d 7 in WT neutrophil-transplanted and *miR-223*$^{Y/-}$ neutrophil-transplanted wounds (n = 6).

E. Measurement of the granulation tissue area at d 14 wound sites from WT neutrophil-transplanted and *miR-223*$^{Y/-}$ neutrophil-transplanted wounds (n = 6).

Data information: All values represent the mean ± SD. Unpaired *t*-tests were used to generate *P*-values indicated in the Figure. *$P<0.05$.

Appendix Table S1. Number of annotated reads at various time points in skin wound sites.

Category	Intact	Day 1	Day 3	Day 7	Day 10	Day 14
total_reads	2,448,759	2,172,903	1,499,496	2,179,215	2,031,016	2,338,879
filtered_out_by_trim	92,478	135,833	105,836	74,749	86,501	87,952
filtered_pass	2,356,281	2,037,070	1,393,660	2,104,466	1,944,515	2,250,927
genome_aligned	2,334,669	2,019,622	1,386,056	2,094,861	1,909,111	2,239,740
target_miRNA	1,920,358 (82.3%)	1,776,639 (88.0%)	1,219,212 (88.0%)	1,924,139 (91.9%)	1,462,271 (76.6%)	2,038,726 (91.0%)
other_miRNA	6,679 (0.3%)	5,210 (0.3%)	4,135 (0.3%)	5,187 (0.2%)	6,209 (0.3%)	5,845 (0.3%)
piRNA	51,457 (2.2%)	18,437 (0.9%)	13,974 (1.0%)	10,887 (0.5%)	51,115 (2.7%)	11,480 (0.5%)
rRNA	12,620 (0.5%)	2,354 (0.1%)	2,302 (0.2%)	2,838 (0.1%)	11,169 (0.6%)	3,259 (0.1%)
tRNA	757 (0.03%)	254 (0.01%)	28 (0.0002%)	371 (0.02%)	330 (0.02%)	485 (0.02%)
snRNA	3,443 (0.1%)	6,197 (0.3%)	5,628 (0.4%)	1,313 (0.1%)	5,108 (0.3%)	1,465 (0.1%)
snoRNA	3,177 (0.1%)	2,245 (0.1%)	996 (0.1%)	815 (0.04%)	2,218 (0.1%)	933 (0.04%)
scRNA	0	0	0	0	0	0
miscRNA	2,120 (0.1%)	606 (0.03%)	368 (0.03%)	514 (0.02%)	1,665 (0.1%)	723 (0.03%)
RefSeq transcripts	193,247 (8.3%)	84,105 (4.2%)	43,293 (3.1%)	63,332 (3.0%)	229,026 (12.0%)	79,483 (3.5%)
Only Genome	140,811 (6.0%)	123,575 (6.1%)	96,120 (6.9%)	85,465 (4.1%)	140,000 (7.3%)	97,341 (4.3%)

Abbreviations: piRNA, Piwi-interacting RNA; rRNA, ribosomal RNA; tRNA, transfer RNA; snRNA, small nuclear RNA; snoRNA, small nucleolar RNA; scRNA, small cytoplasmic RNA; miscRNA, miscellaneous other RNA.

*Values in parentheses show the percentage of similarity for each genome aligned sample.

Appendix Table S2. Top 9 candidate inflammation-related miRNA reads at various time points in skin wound sites.

miRNA	Intact	Day 1	Day 3	Day 7	Day 10	Day 14	Day 1 / Intact
mmu-miR-147	6	442	96	8	12	8	73.67
mmu-miR-223	3,957	138,618	29,135	7,251	2,968	5,883	35.03
mmu-miR-129-3p	19	582	64	47	47	54	30.63
mmu-miR-139-5p	575	7,693	1,331	1,051	859	1,114	13.38
mmu-miR-21*	256	2,751	1,721	76	67	34	10.75
mmu-miR-340-5p	250	1,672	1,331	462	179	439	6.69
mmu-miR-142-3p	1,872	12,270	6,540	5,416	2,617	4,324	6.55
mmu-miR-142-5p	2,493	15,944	8,980	5,492	3,146	3,254	6.40
mmu-miR-486	1,834	8,266	3,040	5,010	4,851	4,243	4.51

Abbreviation: mmu, *Mus musculus*. Note: Cutoff value > 4.5 data from Dataset EV1.

Appendix Table S3. High expression of miRNA reads at various time points in skin wound sites.

Intact skin vs. day 3

miRNA	Intact	Day 1	Day 3	Day 7	Day 10	Day 14	Day 3 / Intact
mmu-miR-147	6	442	96	8	12	8	16.00
mmu-miR-223	3957	138618	29135	7251	2968	5883	7.36
mmu-miR-21*	256	2751	1721	76	67	34	6.72
mmu-miR-450b-5p	2	13	12	33	8	20	6.00
mmu-miR-18b	3	7	16	2	3	3	5.33
mmu-miR-340-5p	250	1672	1331	462	179	439	5.32
mmu-miR-21	46385	108302	218138	400610	195053	267573	4.70
mmu-miR-363	7	57	32	38	14	27	4.57

Note: Cutoff value > 4.5 data from Dataset EV1.

Intact skin vs. day 7

miRNA	Intact	Day 1	Day 3	Day 7	Day 10	Day 14	Day 7 / Intact
mmu-miR-592	2	5	8	54	66	24	27.00
mmu-miR-155	23	18	23	409	302	307	17.78
mmu-miR-450b-5p	2	13	12	33	8	20	16.50
mmu-miR-335-3p	9	3	2	146	36	46	16.22
mmu-miR-297a	1	1	2	13	9	6	13.00
mmu-miR-214*	84	109	104	900	611	973	10.71
mmu-miR-672	4	0	0	42	19	9	10.50
mmu-miR-29a*	14	25	17	134	123	226	9.57
mmu-miR-450a-5p	210	207	179	1913	581	1177	9.11
mmu-miR-1188	4	4	8	36	24	10	9.00
mmu-miR-21	46385	108302	218138	400610	195053	267573	8.64
mmu-miR-574-5p	4	6	2	29	30	45	7.25
mmu-miR-455*	19	7	11	136	87	130	7.16
mmu-miR-137	1	5	2	7	3	9	7.00

mmu-miR-294	1	1	2	7	1	8	7.00
mmu-miR-499	4	4	6	27	4	14	6.75
mmu-miR-27a*	2	13	0	12	6	9	6.00
mmu-miR-875-5p	1	0	0	6	1	5	6.00
mmu-miR-126-5p	1244	632	498	7372	6015	8831	5.93
mmu-miR-136	1197	1034	522	7008	4893	7569	5.85
mmu-miR-679	3	5	3	17	10	9	5.67
mmu-miR-667	60	56	55	329	299	224	5.48
mmu-miR-34b-3p	33	81	113	180	103	83	5.45
mmu-miR-363	7	57	32	38	14	27	5.43
mmu-miR-467c	5	16	4	23	19	16	4.60
mmu-miR-467d	5	0	2	23	19	20	4.60

Note: Cutoff value > 4.5 data from Dataset EV1.

Intact skin vs. day 10

miRNA	Intact	Day 1	Day 3	Day 7	Day 10	Day 14	Day 10 / Intact
mmu-miR-592	2	5	8	54	66	24	33.00
mmu-miR-155	23	18	23	409	302	307	13.13
mmu-miR-297a	1	1	2	13	9	6	9.00
mmu-miR-29a*	14	25	17	134	123	226	8.79
mmu-miR-574-5p	4	6	2	29	30	45	7.50
mmu-miR-214*	84	109	104	900	611	973	7.27
mmu-miR-1934	1	10	1	4	7	5	7.00
mmu-miR-206	3585	8831	5407	15022	23863	14210	6.66
mmu-miR-1188	4	4	8	36	24	10	6.00
mmu-miR-466f-3p	2	22	3	4	12	5	6.00
mmu-miR-384-5p	1	0	0	3	5	2	5.00
mmu-miR-701	2	0	5	7	10	7	5.00
mmu-miR-667	60	56	55	329	299	224	4.98

mmu-miR-126-5p	1244	632	498	7372	6015	8831	4.84
mmu-miR-672	4	0	0	42	19	9	4.75
mmu-miR-455*	19	7	11	136	87	130	4.58
mmu-miR-297c	2	8	1	4	9	9	4.50

Note: Cutoff value > 4.5 data from Dataset EV1.

Intact skin vs. day 14

miRNA	Intact	Day 1	Day 3	Day 7	Day 10	Day 14	Day 14 / Intact
mmu-miR-29a*	14	25	17	134	123	226	16.14
mmu-miR-155	23	18	23	409	302	307	13.35
mmu-miR-592	2	5	8	54	66	24	12.00
mmu-miR-214*	84	109	104	900	611	973	11.58
mmu-miR-574-5p	4	6	2	29	30	45	11.25
mmu-miR-450b-5p	2	13	12	33	8	20	10.00
mmu-miR-137	1	5	2	7	3	9	9.00
mmu-miR-294	1	1	2	7	1	8	8.00
mmu-miR-126-5p	1244	632	498	7372	6015	8831	7.10
mmu-miR-190b	1	1	0	1	3	7	7.00
mmu-miR-455*	19	7	11	136	87	130	6.84
mmu-miR-136	1197	1034	522	7008	4893	7569	6.32
mmu-miR-297a	1	1	2	13	9	6	6.00
mmu-miR-21	46385	108302	218138	400610	195053	267573	5.77
mmu-miR-450a-5p	210	207	179	1913	581	1177	5.60
mmu-miR-9	12	12	10	44	24	65	5.42
mmu-miR-124	9	19	13	23	39	46	5.11
mmu-miR-335-3p	9	3	2	146	36	46	5.11
mmu-miR-146a	1394	2833	3633	5372	4318	7019	5.04
mmu-miR-1934	1	10	1	4	7	5	5.00
mmu-miR-875-5p	1	0	0	6	1	5	5.00

mmu-miR-27a*	2	13	0	12	6	9	4.50
mmu-miR-297c	2	8	1	4	9	9	4.50

Note: Cutoff value > 4.5 data from Dataset EV1.

Appendix Table S4. The profile of each human sample.

Sex	Age (years)	Etiology	Biopsy site
M	67	Epidermal cyst	Face
M	75	Cellulitis with microabscess	Face
M	60	Inflammatory granulation tissue with microabscess	Abdominal
F	32	Cystic lesion with inflammatory granulation tissue and fibrosis	Inguinal
F	79	Abscess	Face
F	71	Epidermal cyst	Neck
M	28	Epidermal cyst with microabscess and inflammatory granulation tissue	Face
M	82	Necrotizing lesion with neutrophil and eosinophil infiltration	Head
F	56	Epidermal cyst with multiple abscess and chrnic inflammation and psoriasiform dermatitis	Hip
F	12	Abscess forming granulomatous inflammation	Eyelid
F	23	Cystic lesion with inflammatory granulation tissue, foreign body reaction, and granuloma	Arm
F	33	Pustular dermatitis with erosion, ulcer, and gram positive cocci	Arm
F	47	Dermal neutrophilic infiltration	Thing
F	75	Abscess	Leg
M	62	Cutaneous ulcer	Foot

Abbreviations: M, Male; F, Female

Appendix Table S5. List of antibodies.

Primary antibody	Company	Catalog No.	Species	Dilution
α smooth muscle actin	Abcam	ab5694	Rabbit	1:500
F4/80	Abcam	ab6640	Rat	1:400
IL6	Abcam	ab6672	Rabbit	1:400
Neutrophil (Ly-6G and Ly-6C)	Abcam	ab2557	Rat	1:400
Alexa Fluor 568	ThermoFisher	A-11011	Goat	1:1000
DIG	Roche Diagnostics	11093274910	Sheep	1:800
Ago2	Wako Pure Chemical Industries	292-67301	Mouse	5 μg/IP
C/EBPα	GeneTex	GTX100674	Rabbit	5 μg/IP
Histone H3	Cell Signaling Technology	4620	Rabbit	5 μg/IP
Normal rabbit IgG	Cell Signaling Technology	2729	Rabbit	5 μg/IP

Abbreviation: IP, immunoprecipitation.

Appendix Table S6. List of primers.

Gene symbol	Sequences (5′-3′)
Rps18	F) 5′-TTCTGGCCAACGGTCTAGACAAC-3′<br>(R) 5′-CCAGTGGTCTTGGTGTGCTGA-3′
Il6 (M)	(F) 5′-CAACGATGATGCACTTGCAGA-3′<br>(R) 5′-CTCCAGGTAGCTATGGTACTCCAGA-3′
Il6ra	(F) 5′- CCTGAGACTCAAGCAGAAATGG-3′<br>(R) 5′-AGAAGGAAGGTCGGCTTCAGT-3′
Il6st	(F) 5′- CCGTGTGGTTACATCTACCCT-3′<br>(R) 5′-CGTGGTTCTGTTGATGACAGTG-3′
Il1b	(F) 5′-TCCAGGATGAGGACATGAGCAC-3′<br>(R) 5′-GAACGTCACACACCAGCAGGTTA-3′
B2M	(F) 5′-CGGGCATTCCTGAAGCTGA-3′<br>(R) 5′-GGATGGATGAAACCCAGACACATAG-3′
IL6 (H)	(F) 5′-AAGCCAGAGCTGTGCAGATGAGTA-3′<br>(R) 5′-TGTCCTGCAGCCACTGGTTC-3′
CEBPA	(F) 5′-TCGTGGGTCAGCTCTGAGGA-3′<br>(R) 5′-GCAATGCTGAAGGCATACAGTACAA-3′
RUNX1	(F) 5′-CACTGGCGCTGCAACAAGA-3′<br>(R) 5′-CCAGCCATCACAGTGACCAGA-3′
SPI1	(F) 5′-GCCCTATGACACGGATCTATACCAA-3′<br>(R) 5′-TCTCGGCGAAGCTCTCGAA-3′
ChIP-PCR<br>ChIP-qPCR	(F) 5′-GCCCTCTTTGTTGATGTGTC-3′<br>(R) 5′-GGCAGCTATTAAAGTGCCCT-3′

Abbreviations: F; forward, R; reverse, M; *Mus musculus*, H; *Homo sapiens.*

Appendix Table S7. Statistical analysis.

Fig 1. *miR-147*

Group compared	Summary	P value	Statistical test
Intact vs. d 1	****	< 0.0001	Tukey's multiple comparisons test
Intact vs. d 3	***	0.0005	Tukey's multiple comparisons test
d 1 vs. d 7	****	< 0.0001	Tukey's multiple comparisons test
d 3 vs. d 7	***	0.0005	Tukey's multiple comparisons test

Fig 1. *miR-223*

Group compared	Summary	P value	Statistical test
Intact vs. d 1	****	< 0.0001	Tukey's multiple comparisons test
Intact vs. d 3	**	0.0011	Tukey's multiple comparisons test
d 1 vs. d 3	**	0.0029	Tukey's multiple comparisons test
d 1 vs. d 7	****	< 0.0001	Tukey's multiple comparisons test

Fig 1. *miR-129-3p*

Group compared	Summary	P value	Statistical test
Intact vs. d 1	*	0.0461	Tukey's multiple comparisons test
Intact vs. d 3	****	< 0.0001	Tukey's multiple comparisons test
d 1 vs. d 3	***	0.0001	Tukey's multiple comparisons test
d 3 vs. d 7	****	< 0.0001	Tukey's multiple comparisons test

Fig 1. ***miR-139-5p***

Group compared	Summary	P value	Statistical test
Intact vs. d 1	****	< 0.0001	Tukey's multiple comparisons test
Intact vs. d 3	***	0.0005	Tukey's multiple comparisons test
Intact vs. d 7	*	0.0252	Tukey's multiple comparisons test
d 1 vs. d 3	***	0.0009	Tukey's multiple comparisons test
d 1 vs. d 7	****	< 0.0001	Tukey's multiple comparisons test

Fig 1. ***miR-21****

Group compared	Summary	P value	Statistical test
Intact vs. d 1	**	0.0056	Tukey's multiple comparisons test
d 1 vs. d 3	*	0.0440	Tukey's multiple comparisons test
d 1 vs. d 7	*	0.0470	Tukey's multiple comparisons test

Fig 1. ***miR-340-5p***

Group compared	Summary	P value	Statistical test
Intact vs. d 1	**	0.0070	Tukey's multiple comparisons test
Intact vs. d 3	****	0.0005	Tukey's multiple comparisons test
d 1 vs. d 3	***	0.0001	Tukey's multiple comparisons test
d 3 vs. d 7	****	< 0.0001	Tukey's multiple comparisons test

Fig 1. *miR-142-3p*

Group compared	Summary	P value	Statistical test
Intact vs. d 1	*	0.0486	Tukey's multiple comparisons test
Intact vs. d 3	**	0.0027	Tukey's multiple comparisons test
d 3 vs. d 7	**	0.0080	Tukey's multiple comparisons test

Fig 1. *miR-142-5p*

Group compared	Summary	P value	Statistical test
Intact vs. d 1	****	< 0.0001	Tukey's multiple comparisons test
Intact vs. d 3	****	< 0.0001	Tukey's multiple comparisons test
d 1 vs. d 7	****	< 0.0001	Tukey's multiple comparisons test
d 3 vs. d 7	****	< 0.0001	Tukey's multiple comparisons test

Fig 2A. Expression of *miR-223*

Group compared	Summary	P value	Statistical test
Intact vs. 12 hrs	****	< 0.0001	Tukey's multiple comparison test
0.5hr vs. 12 hrs	****	< 0.0001	Tukey's multiple comparison test
3 hrs vs. 12 hrs	**	0.0062	Tukey's multiple comparison test
12 hrs vs. 24 hrs	***	0.0003	Tukey's multiple comparison test

Fig 2A. Expression of *miR-142-3p*

Group compared	Summary	P value	Statistical test
Intact vs. 12 hrs	**	0.0032	Tukey's multiple comparison test
0.5hr vs. 3 hrs	*	0.0500	Tukey's multiple comparison test
0.5hr vs. 12 hrs	**	0.0032	Tukey's multiple comparison test

Fig 2B. Expression of *miR-223*

Group compared	Summary	P value	Statistical test
WT vs. PU.1$^{+/-}$	****	< 0.0001	Tukey's multiple comparison test
WT vs. PU.1$^{-/-}$	****	< 0.0001	Tukey's multiple comparison test
PU.1$^{+/-}$ vs. PU.1$^{-/-}$	***	0.0004	Tukey's multiple comparison test

Fig 2B. Expression of *miR-142-3p*

Group compared	Summary	P value	Statistical test
WT vs. PU.1$^{+/-}$	****	< 0.0001	Tukey's multiple comparison test
WT vs. PU.1$^{-/-}$	****	< 0.0001	Tukey's multiple comparison test
PU.1$^{+/-}$ vs. PU.1$^{-/-}$	***	0.0004	Tukey's multiple comparison test

Fig 2B. Expression of *miR-142-5p*

Group compared	Summary	P value	Statistical test
WT vs. *PU.1*$^{+/-}$	****	< 0.0001	Tukey's multiple comparison test
WT vs. *PU.1*$^{-/-}$	****	< 0.0001	Tukey's multiple comparison test
PU.1$^{+/-}$ vs. *PU.1*$^{-/-}$	**	0.0024	Tukey's multiple comparison test

Fig 2B. Expression of *miR-139-5p*

Group compared	Summary	P value	Statistical test
WT vs. *PU.1*$^{-/-}$	**	0.0064	Tukey's multiple comparison test
PU.1$^{+/-}$ vs. *PU.1*$^{-/-}$	**	0.0028	Tukey's multiple comparison test

Fig 3F. WT vs. *miR-223*$^{Y/-}$

Group compared	Summary	P value	Statistical test
3 d	***	0.0003	Sidak's multiple comparisons test
7 d	*	0.0376	Sidak's multiple comparisons test

Fig 3H. WT vs. *miR-223*$^{Y/-}$

Group compared	Summary	P value	Statistical test
3 d	*	0.0115	Unpaired Student's *t*-tests

Fig 3J. WT vs. *miR-223*$^{Y/-}$

Group compared	Summary	P value	Statistical test
7 d	*	0.0206	Unpaired Student's *t*-tests
14 d	*	0.0246	Unpaired Student's *t*-tests

Fig 4B. WT vs. *miR-223*$^{Y/-}$

Group compared	Summary	P value	Statistical test
3 h	*	0.0445	Unpaired Student's *t*-tests
3 d	*	0.0445	Sidak's multiple comparisons test

Fig 4D. WT vs. *miR-223*$^{Y/-}$

Group compared	Summary	P value	Statistical test
1 d	*	0.0322	Unpaired Student's *t*-tests
3 d	*	0.0025	Unpaired Student's *t*-tests

Fig 4E. WT vs. *miR-223*$^{Y/-}$

Group compared	Summary	P value	Statistical test
1 d	*	0.0285	Unpaired Student's *t*-tests
3 d	*	0.0101	Unpaired Student's *t*-tests

Fig 4G. WT vs. *miR-223*$^{Y/-}$

Group compared	Summary	P value	Statistical test
17 min	*	0.0356	Mann Whitney test
45 min	*	0.0356	Mann Whitney test
46 min	*	0.0415	Mann Whitney test
47 min	*	0.0259	Mann Whitney test
48 min	*	0.0167	Mann Whitney test
49 min	*	0.0156	Mann Whitney test
50 min	*	0.0151	Mann Whitney test
51 min	*	0.0173	Mann Whitney test
52 min	*	0.0146	Mann Whitney test
53 min	*	0.0162	Mann Whitney test
54 min	*	0.0141	Mann Whitney test

55 min	*	0.0173	Mann Whitney test
56 min	*	0.0146	Mann Whitney test
57 min	*	0.0156	Mann Whitney test
58 min	*	0.0131	Mann Whitney test
59 min	*	0.0151	Mann Whitney test
60 min	*	0.0113	Mann Whitney test

Fig 5B. WT vs. *miR-223*$^{Y/-}$

Group compared	Summary	P value	Statistical test
Il6	*	0.0245	Unpaired Student's *t*-tests

Fig 5H. *miR-223* mimic vs. each sample

Group compared	Summary	P value	Statistical test
vs. control	**	0.0093	Dunnett's multiple comparisons test
vs. mutation 2	*	0.0316	Dunnett's multiple comparisons test
vs. mutation 3	**	0.0064	Dunnett's multiple comparisons test

Fig 6B. WT vs. *miR-223*$^{Y/-}$

Group compared	Summary	P value	Statistical test
3 d	*	0.0467	Sidak's multiple comparisons test
7 d	**	0.0011	Sidak's multiple comparisons test

Fig 6C. WT vs. *miR-223*$^{Y/-}$

Group compared	Summary	P value	Statistical test
3d	*	0.0403	Unpaired Student's *t*-tests

Fig 6E. WT vs. *miR-223*$^{Y/-}$

Group compared	Summary	P value	Statistical test
1d	*	0.0403	Unpaired t test with Welch's correction

Fig 6F. WT vs. *miR-223*$^{Y/-}$

Group compared	Summary	P value	Statistical test
Il6	*	0.0394	Unpaired test
Il6ra	**	0.0013	Unpaired Student's *t*-tests
Il1β	*	0.0371	Unpaired t test with Welch's correction

Fig 6H. 3d

Group compared	Summary	P value	Statistical test
WT control vs. WT-neutrophil	-	0.4992	Tukey's multiple comparisons test
WT control vs. KO-neutrophil	*	0.0124	Tukey's multiple comparisons test
WT neutrophil vs. KO-neutrophil	-	0.1961	Tukey's multiple comparisons test

Fig 6H. 7d

Group compared	Summary	P value	Statistical test
WT control vs. WT-neutrophil	-	0.7993	Tukey's multiple comparisons test
WT control vs. KO-neutrophil	**	0.0023	Tukey's multiple comparisons test
WT neutrophil vs. KO-neutrophil	***	0.0002	Tukey's multiple comparisons test

Fig 7B.

Group compared	Summary	P value	Statistical test
Control ODN vs. *miR-223* AS ODN	****	< 0.0001	Unpaired Student's *t*-tests

Fig 7D. Control ODN vs. *miR-223* AS ODN

Group compared	Summary	P value	Statistical test
6 h	**	0.0083	Unpaired t test with Welch's correction
1 d	****	< 0.0001	Unpaired Student’s *t*-tests

Fig 7E.

Group compared	Summary	P value	Statistical test
Control ODN vs. *miR-223* AS ODN	*	0.0307	Unpaired Student’s *t*-tests

Fig 7G. Control ODN vs. *miR-223* AS ODN

Group compared	Summary	P value	Statistical test
3 d	***	0.0002	Sidak’s multiple comparisons test
7 d	**	0.0064	Sidak’s multiple comparisons test

Fig 7H. Control ODN vs. *miR-223* AS ODN

Group compared	Summary	P value	Statistical test
Wound	*	0.0217	Unpaired t test with Welch's correction
Necrosis	*	0.0309	Unpaired t test with Welch's correction

Fig 7I.

Group compared	Summary	P value	Statistical test
Control ODN vs. *miR-223* AS ODN	**	0.0010	Unpaired Student’s *t*-tests

Fig 7J.

Group compared	Summary	P value	Statistical test
Control ODN vs. *miR-223* AS ODN	***	0.0005	Unpaired Student’s *t*-tests

Fig 8A. Expression of *miR-223* in HL-60 cells

**Group compared**	**Summary**	**P value**	**Statistical test**
Control vs. 2 d	*	0.0499	Holm-Sidak's multiple comparisons test
Control vs. 3 d	*	0.0137	Holm-Sidak's multiple comparisons test
Control vs. 4 d	****	< 0.0001	Holm-Sidak's multiple comparisons test
Control vs. 5 d	****	< 0.0001	Holm-Sidak's multiple comparisons test

Fig 8B. *miR-223* AS ODN *miR-223* expression in dHL60

**Group compared**	**Summary**	**P value**	**Statistical test**
Control vs. *miR-223* AS ODN	*	0.0385	Unpaired t test with Welch's correction
Control ODN vs. *miR-223* AS ODN	*	0.0384	Unpaired t test with Welch's correction

Fig 8C. Non PGN vs. PGN

**Group compared**	**Summary**	**P value**	**Statistical test**
Control	*	0.0176	Unpaired Student's *t*-tests
miR-223 AS ODN	**	0.0056	Unpaired Student's *t*-tests

Fig 8D. Control ODN vs. *miR-223* AS ODN

**Group compared**	**Summary**	**P value**	**Statistical test**
Non PGN	**	0.0056	Unpaired Student's *t*-tests
PGN	**	0.0096	Unpaired Student's *t*-tests

Fig 8E. Control ODN vs. *miR-223* AS ODN

Group compared	Summary	P value	Statistical test
Non PGN	**	0.0089	Unpaired Student's *t*-tests
6 hrs	*	0.0120	Unpaired Student's *t*-tests
12 hrs	*	0.0116	Unpaired Student's t-tests

Fig 8G. Relative binding activity

Group compared	Summary	P value	Statistical test
Control vs. PGN	*	0.0361	Unpaired Student's *t*-tests

Fig EV1C. WT vs. *miR-223*$^{Y/-}$

Group compared	Summary	P value	Statistical test
d 7	*	0.0206	Unpaired t test
d 14	*	0.0246	Unpaired t test

Fig EV1E.

Group compared	Summary	P value	Statistical test
WT vs. *miR-223*$^{Y/-}$	**	0.0013	Unpaired t test

Fig EV2B. WT vs. *miR-223*$^{Y/-}$

Group compared	Summary	P value	Statistical test
d 3	**	0.0044	Unpaired t test
d 7	*	0.0154	Unpaired t test

Fig EV2C.

Group compared	Summary	P value	Statistical test
WT vs. *miR-223*$^{Y/-}$	*	0.0478	Unpaired t test

Fig EV3C. WT vs. *miR-223*$^{Y/-}$

Group compared	Summary	P value	Statistical test
d 3	*	0.0206	Unpaired t test
d 7	*	0.0367	Unpaired t test

Fig EV3D. WT vs. *miR-223*$^{Y/-}$

Group compared	Summary	P value	Statistical test
Wound	*	0.0467	Unpaired t test
Necrosis	*	0.0434	Unpaired t test

Fig EV3F.

Group compared	Summary	P value	Statistical test
WT vs. *miR-223*$^{Y/-}$	*	0.0470	Unpaired t test

Fig EV4A. Control ODN vs. miR-223 AS ODN

Group compared	Summary	P value	Statistical test
d 7	***	0.0004	Sidak’s multiple comparisons test

Fig EV4B. Pluronic gel vs. PB gel

Group compared	Summary	P value	Statistical test
d 3	**	0.0012	Sidak’s multiple comparisons test

Fig EV5A. Non PGN vs. PGN

Group compared	Summary	P value	Statistical test
CEBPA	**	0.0089	Unpaired t test

Appendix Fig S4C. WT neutrophil vs. *miR-223*$^{Y/-}$ neutrophil

Group compared	Summary	P value	Statistical test
Wound	*	0.0467	Unpaired t test
Necrosis	-	0.0933	Unpaired t test
Granulation tissue	*	0.0441	Unpaired t test

Appendix Fig S4D.

Group compared	Summary	P value	Statistical test
WT neutrophil vs. *miR-223*$^{Y/-}$ neutrophil	*	0.0282	Unpaired t test

Appendix Fig S4E.

Group compared	Summary	P value	Statistical test
WT neutrophil vs. *miR-223*$^{Y/-}$ neutrophil	*	0.0446	Unpaired t test
