# Supplementary material for: Targeting miR‐223 in neutrophils enhances the clearance of Staphylococcus aureus in infected wounds
Source: EMBO Mol Med. 2018 Aug 31;10(10):e9024. doi: 10.15252/emmm.201809024 (PMC6180296; doi:10.15252/emmm.201809024)
Supplement: Supplementary file 2 — Expanded View Figures PDF [file EMMM-10-e9024-s002.pdf]

Expanded View Figures

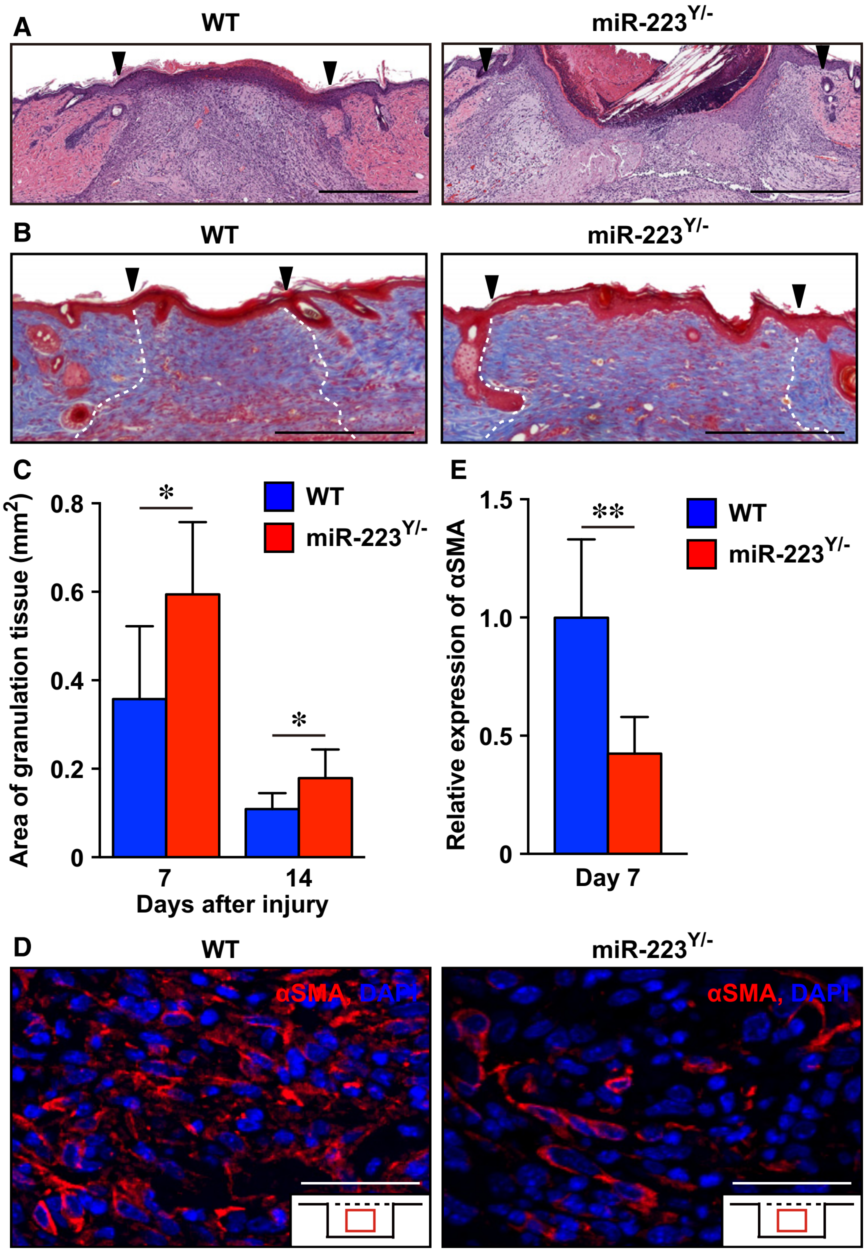

Figure EV1. Attenuation of wound contraction and increased scar area in *miR-223*$^{Y/-}$ mice.

A Representative images using H&E staining of wound sites at day 7 in WT (*n* = 8) and *miR-223*$^{Y/-}$ (*n* = 6) mice. Scale bars: 500 μm.

B Representative images of Masson's Trichrome staining of collagen fibers (blue) in skin wound tissues of WT (*n* = 7) and *miR-223*$^{Y/-}$ (*n* = 8) mice at day 14 after injury. Scar sites were visualized at the mid-point of the wound (indicated by dotted line). Nuclei are stained black, and cytosol is stained red. Scar formation is clearly recognizable by day 14 after injury. Scale bars: 500 μm.

C Measurement of granulation tissue area at wound sites from WT (day 7; *n* = 8, day 14; *n* = 7) and *miR-223*$^{Y/-}$ (day 7; *n* = 6, day 14; *n* = 8) mice.

D Representative images of αSMA-positive wound-infiltrated myofibroblasts at day 7 in wound sites from WT and *miR-223*$^{Y/-}$ mice (*n* = 7). Scale bars: 50 μm.

E Quantification of expression of αSMA at day 7 in wound sites from WT and *miR-223*$^{Y/-}$ mice (*n* = 7).

Data information: All values represent the mean ± SD. Unpaired *t*-tests were used to generate *P*-values indicated in the Figure. $^{*}P < 0.05$, $^{**}P < 0.01$.

 © 2018 The Authors

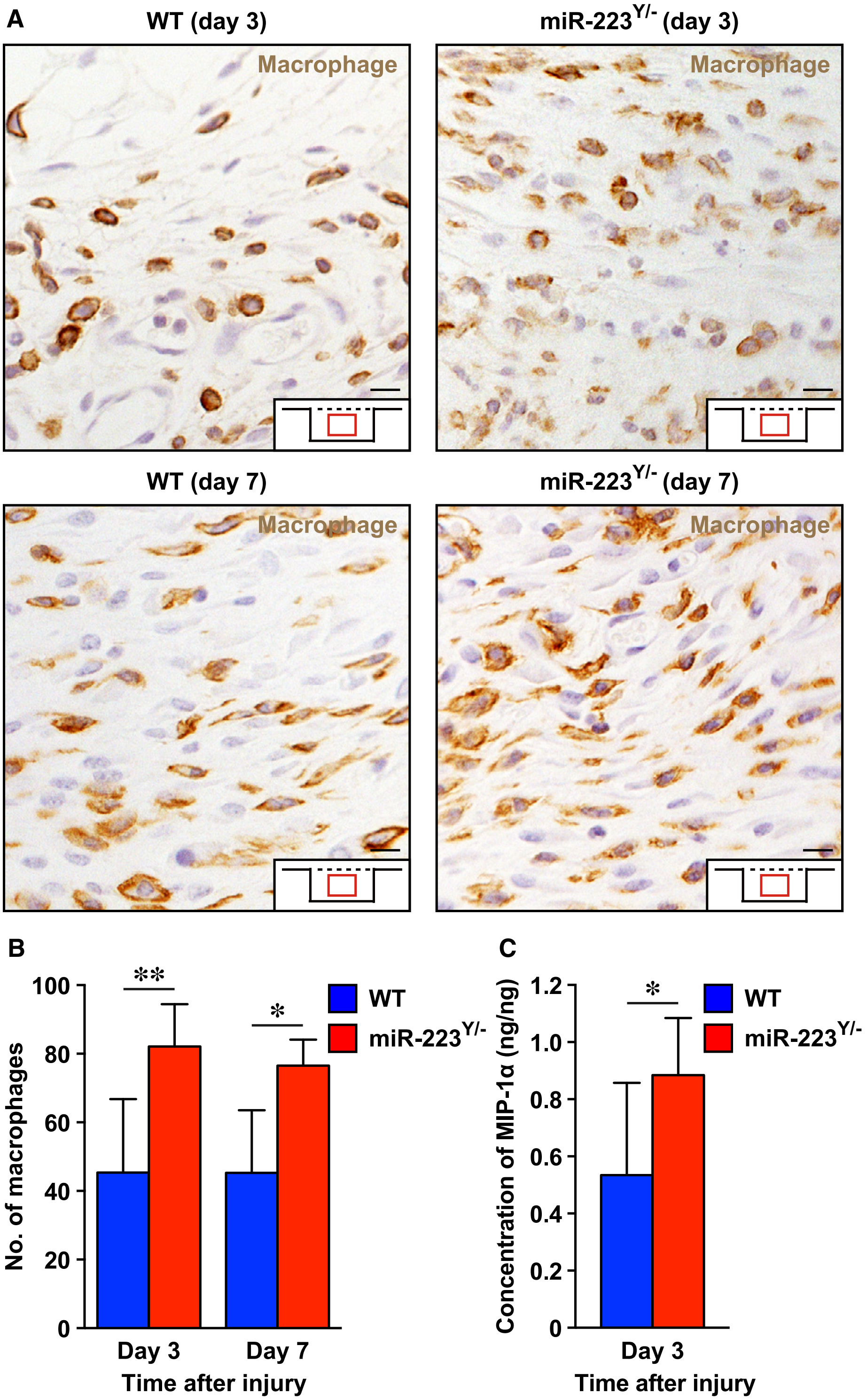

Figure EV2. Macrophage infiltration of wound sites in *miR-223*$^{Y/-}$ and WT mice.

A Representative images of F4/80-positive macrophages at wound sites from WT (day 3; n = 6, day 7; n = 5) and *miR-223*$^{Y/-}$ (day 3; n = 6, day 7; n = 4) mice. Scale bars: 50 μm.

B Quantification of F4/80-positive macrophages at wound sites from WT (day 3; n = 6, day 7; n = 5) and *miR-223*$^{Y/-}$ (day 3; n = 6, day 7; n = 4) mice.

C Measurements of MIP-1α at wound sites using ELISA (n = 6).

Data information: All values represent the mean ± SD. Unpaired t-tests were used to generate P-values indicated in the Figure. *P < 0.05, **P < 0.01.

© 2018 The Authors

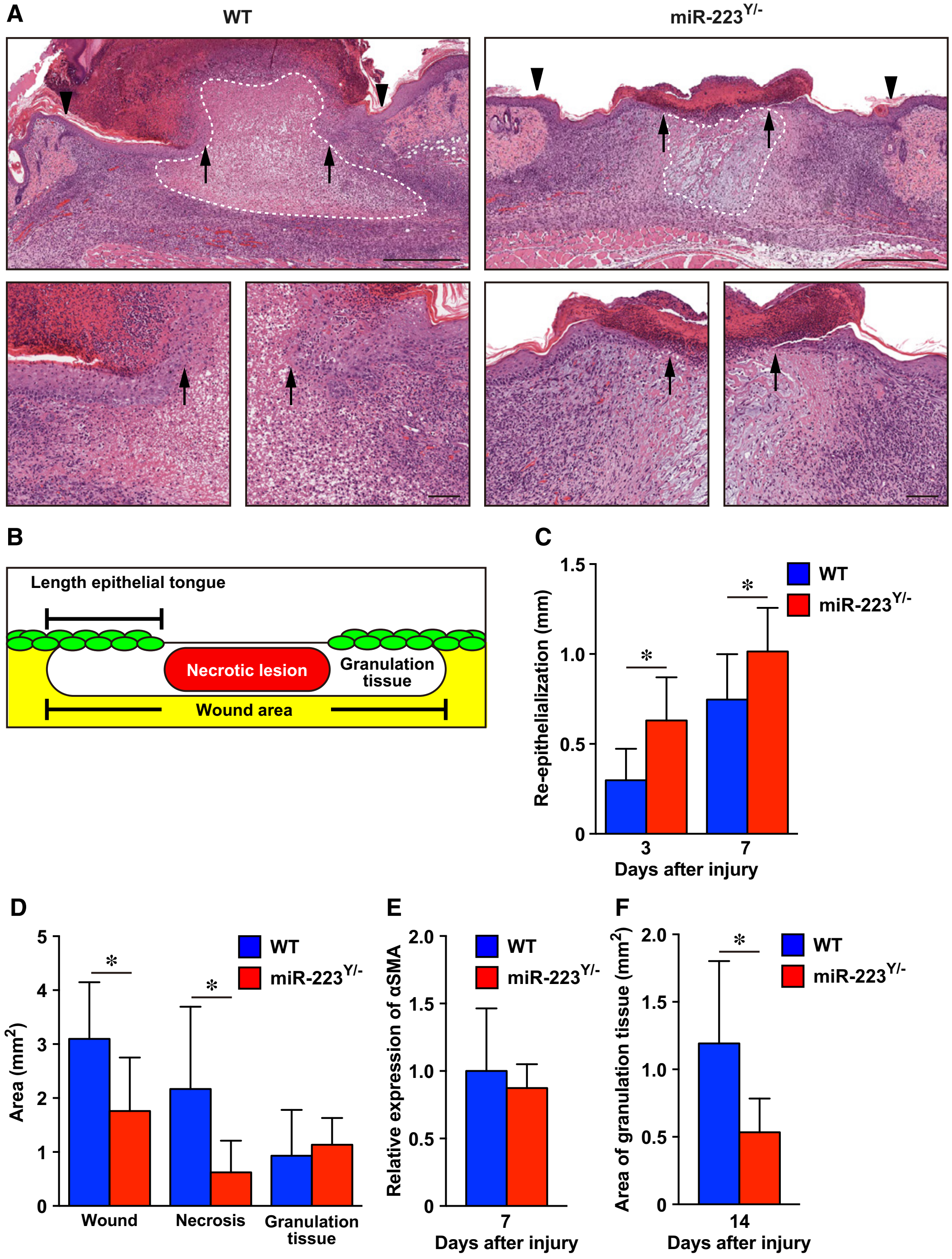

Figure EV3. *miR-223*$^{Y/-}$ mice show accelerated *Staphylococcus aureus*-infected skin wound healing.

A H&E staining of re-epithelialization at day 7 after injury (wound margin (arrowheads) and the leading edge of epithelia (arrows)). Dotted line indicates pathological necrotic lesion. Scale bars: 500 μm (top) and 100 μm (bottom).

B Schematic indicating the measurements derived from histological sections.

C Measurement of the epithelial tongue at day 3 and day 7 after injury in WT (day 3; n = 4, day 7; n = 8) and *miR-223*$^{Y/-}$ (day 3; n = 5, day 7; n = 6) mice.

D Measurement of the total wound area, necrotic lesion area, and granulation tissue area at day 7 after injury in WT neutrophil-transplanted and *miR-223*$^{Y/-}$ neutrophil-transplanted wounds (n = 6).

E Quantification of the expression of αSMA at day 7 in *S. aureus*-infected wound sites from WT (n = 6) and *miR-223*$^{Y/-}$ mice (n = 7).

F Measurements of the granulation tissue area at day 14 in WT (n = 7) or *miR-223*$^{Y/-}$ wound sites (n = 5).

Data information: All values represent the mean ± SD. Unpaired t-tests were used to generate P-values indicated in the Figure. *$P < 0.05$.

© 2018 The Authors

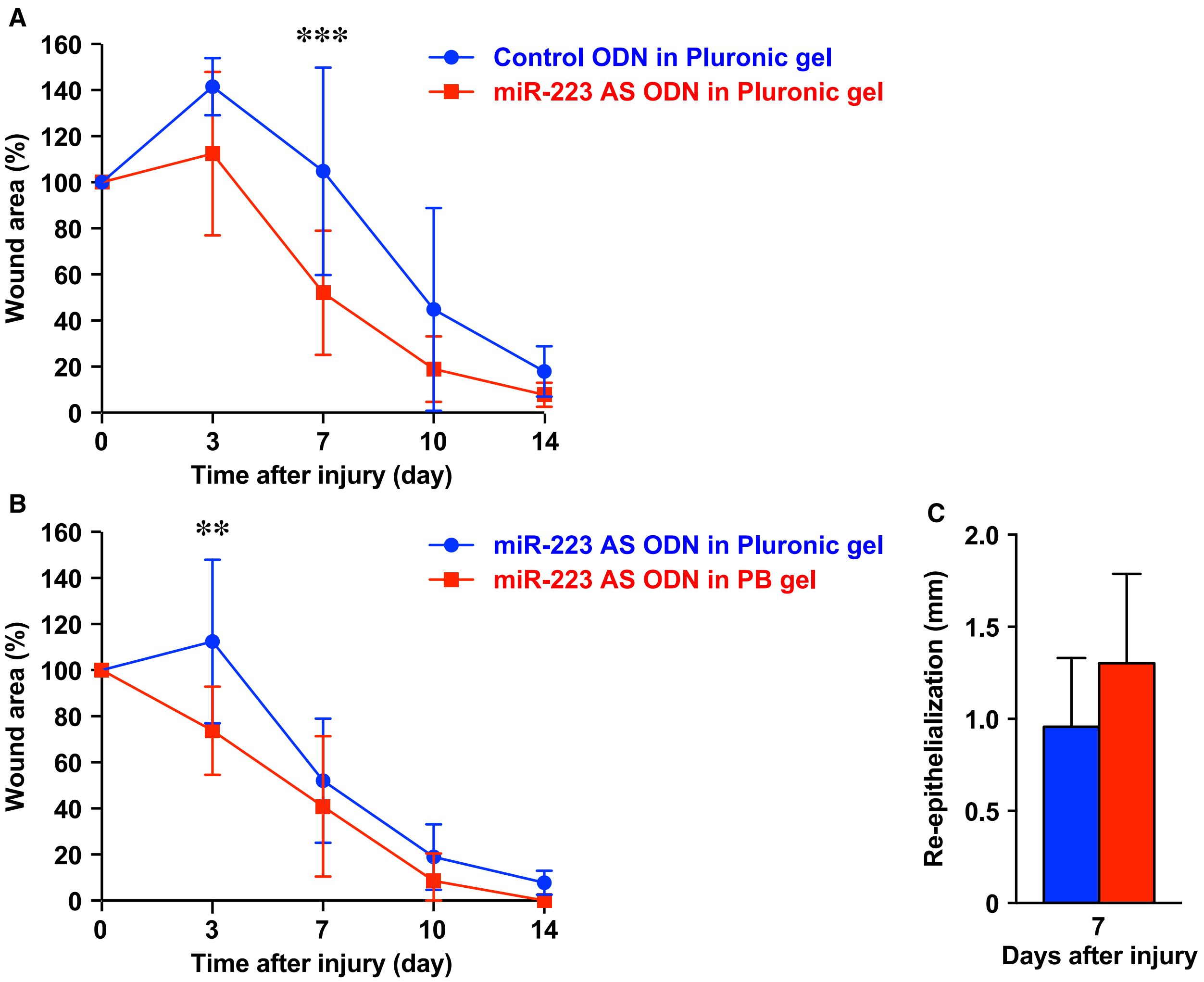

Figure EV4. *miR-223* AS ODN in PB gel-treated *Staphylococcus aureus*-infected skin wound sites are improved compared with Pluronic gel.

A The proportion of the wound remaining open relative to the initial *S. aureus*-infected wound area at each time point after the injury in control ODN in Pluronic gel versus *miR-223* AS ODN in Pluronic gel-treated wounds ($n = 8$).

B The proportion of the wound remaining open relative to the initial *S. aureus*-infected wound area at each time point after injury in PB gel ($n = 7$) versus Pluronic gel ($n = 8$).

C Measurement of the epithelial tongue at day 7 after injury in control ODN in PB gel and *miR-223* AS ODN in PB gel-treated wounds ($n = 6$).

Data information: All values represent the mean ± SD. Two-way ANOVA followed by Sidak multiple comparison test was used to generate the *P*-values indicated in the Figure. $^{**}P < 0.01$. $^{***}P < 0.001$.

© 2018 The Authors

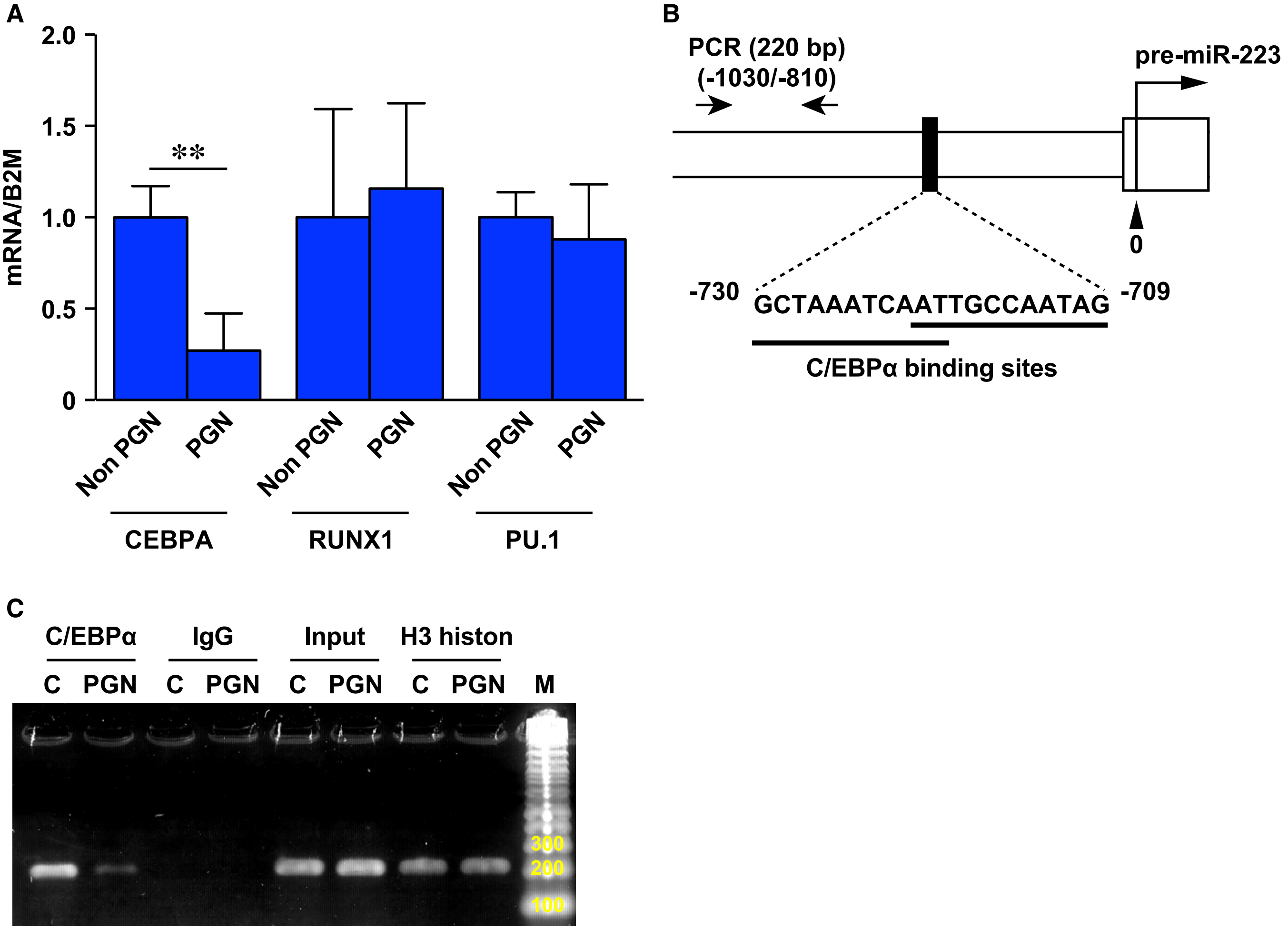

Figure EV5. Expression of *CEBPA*, *RINX1*, and *PU.1*.

A Gene expression of *CEBPA*, *RINX1*, and *PU.1* in dHL-60 after stimulation with PGN for 6 h measured by qPCR relative to beta-2-microglobulin (*B2m*) (n = 3).

B Schematic representation of the genomic composition of the pre-*miR-223* locus and C/EBPα binding sites. ChIP-PCR was performed with PCR primers #1 and #2. The location of primers (−1,030/−810 with respect to the 5′ end of the pre-*miR-223*) is shown in the lower panel together with sequence for the C/EBPα binding sites.

C Representative image of ChIP-PCR production amplified by primers (220 bp) on 2% agarose gel. Anti-C/EBPα Ab-ChIP amplification in non-stimulated dHL-60 cells was markedly increased compared with 6-h PGN-stimulated dHL-60 cells. Control amplification was carried out using anti-IgG isotype control Ab-ChIP (negative control), anti-H3 histone Ab-ChIP (procedure positive control), and input chromatin.

Data information: All values represent the mean ± SD. Unpaired t-tests were used to generate P-values indicated in the Figure. **$P < 0.01$.

 © 2018 The Authors
