# Supplementary material for: Targeting miR‐223 in neutrophils enhances the clearance of Staphylococcus aureus in infected wounds
Source: EMBO Mol Med. 2018 Aug 31;10(10):e9024. doi: 10.15252/emmm.201809024 (PMC6180296; doi:10.15252/emmm.201809024)
Supplement: Supplementary file 5 — Movie EV1 [file EMMM-10-e9024-s005.zip › EMM-2018-09024_MovieEV1/MovieEV1legend.docx]

**Movie EV1. Live *in vitro* fluorescence imaging of ROS production in WT-derived neutrophils**.

APF-loaded WT-derived neutrophils were stimulated with PMA and green fluorescence images were acquired every 1 min for 60 min.
