# Supplementary material for: Targeting miR‐223 in neutrophils enhances the clearance of Staphylococcus aureus in infected wounds
Source: EMBO Mol Med. 2018 Aug 31;10(10):e9024. doi: 10.15252/emmm.201809024 (PMC6180296; doi:10.15252/emmm.201809024)
Supplement: Supplementary file 6 — Movie EV2 [file EMMM-10-e9024-s006.zip › EMM-2018-09024_MovieEV2/MovieEV2legend.docx]

**Movie EV2. Live *in vitro* fluorescence imaging of ROS production in *miR-223^Y/-^*-derived neutrophils.**

APF-loaded *miR-223^Y/-^*-derived neutrophils were stimulated with PMA and green fluorescence images were acquired every 1 min for 60 min
