# Supplementary figures and images for: Targeting miR‐223 in neutrophils enhances the clearance of Staphylococcus aureus in infected wounds
Source: EMBO Mol Med. 2018 Aug 31;10(10):e9024. doi: 10.15252/emmm.201809024 (PMC6180296; doi:10.15252/emmm.201809024)

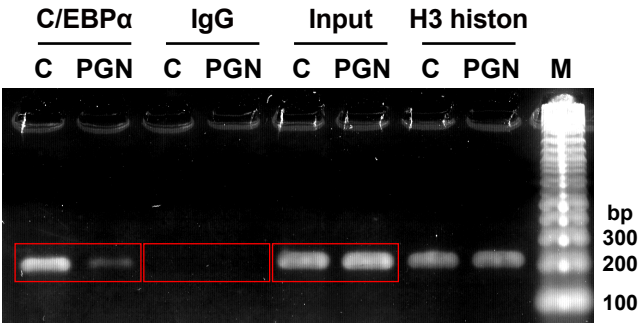

Source Data Figure 8F

Supplement: Supplementary file 7 — Source Data for Expanded View [file EMMM-10-e9024-s007.pdf]
